# Study of binding interaction of rivaroxaban with bovine serum albumin using multi-spectroscopic and molecular docking approach

**DOI:** 10.1186/s13065-017-0366-1

**Published:** 2017-12-20

**Authors:** Tanveer A. Wani, Haitham AlRabiah, Ahmed H. Bakheit, Mohd Abul Kalam, Seema Zargar

**Affiliations:** 1 Department of Pharmaceutical Chemistry, College of Pharmacy, King Saud University, P.O. Box 2457, Riyadh, 11451 Saudi Arabia; 2 Nanomedicine Research Unit, Department of Pharmaceutics, College of Pharmacy, King Saud University, P.O. Box 2457, Riyadh, 11451 Saudi Arabia; 3 Department of Biochemistry, College of Science, King Saud University, PO Box 22452, Riyadh, 11451 Saudi Arabia

**Keywords:** Bovine serum albumin, Rivaroxaban, Human serum albumin, Fluorescence, Quenching

## Abstract

**Background:**

Rivaroxaban is a direct inhibitor of coagulation factor Xa and is used for venous thromboembolic disorders. The rivaroxaban interaction with BSA was studied to understand its PK and PD (pharmacokinetics and pharmacokinetics) properties. Multi-spectroscopic studies were used to study the interaction which included UV spectrophotometric, spectrofluorometric and three dimensional spectrofluorometric studies. Further elucidation of data was done by molecular simulation studies to evaluate the interaction behavior between BSA and rivaroxaban.

**Results:**

Rivaroxaban quenched the basic fluorescence of BSA molecule by the process of static quenching since rivaroxaban and BSA form a complex that results in shift of the absorption spectra of BSA molecule. A decline in the values of binding constants was detected with the increase of temperatures (298–308 K) and the binding constants were in range from 1.32 × 10^5^ to 4.3 × 10^3^ L mol^−1^ indicating the instability of the BSA and rivaroxaban complex at higher temperatures. The data of number of binding sites showed uniformity. The site marker experiments indicated site I (sub-domain IIA) as the principal site for rivaroxaban binding. The thermodynamic study experiments were carried at the temperatures of 298/303/308 K. The ∆G^0^, ∆H^0^ and ∆S^0^ at these temperatures ranged between − 24.67 and − 21.27 kJ mol^−1^ and the values for ∆H^0^ and ∆S^0^ were found to be − 126 kJ mol^−1^ and ∆S − 340 J mol^−1^ K^−1^ The negative value of ∆G^0^ indicating spontaneous binding between the two molecules. The negative values in ∆H^0^ and ∆S^0^ indicated van der Waals interaction and hydrogen bonding were involved during the interaction between rivaroxaban and BSA.

**Conclusions:**

The results of molecular docking were consistent with the results obtained from spectroscopic studies in establishing the principal binding site and type of bonds between rivaroxaban and BSA.

## Background

The serum albumin is most abundant protein in plasma and has high affinity to bind drug ligands and metabolites, thus, acting as a carrier for them. This capability of serum albumin makes it vital to play a function in certain physiological processes such as distribution and transport of various ligands [1, 2]. The ligands bind to albumin either weakly or strongly and the type of binding will have impact on the distribution of these ligands as weakly bound ligands will have poor distribution and fast elimination and the strongly bound ligands will decrease the free ligand amount in plasma. To understand the PK/PD of drug molecules there is a need to investigate the behavior of binding between the drug molecules and albumin [3–11]. Bovine serum albumin (BSA) is structurally analogous to the human serum albumin (HSA) [12], and both of them have been widely studied for their interaction with drug ligands. The studies include multi-spectroscopic and molecular simulation approach with theoretical calculations [13–15].

Rivaroxaban (chemical name 5-chloro-*N*-[[(5S)-2-oxo-3-[4-(3-oxomorpholin-4-yl)phenyl]-1,3-oxazolidin-5-yl]methyl]thiophene-2-carboxamide) inhibits coagulation factor Xa directly and is used for venous thromboembolic disorders. It is prescribed for arthroplasty of hip or knee in adult patients. Conversion of prothrombin to thrombin is catalyzed by factor Xa, thus having a very critical role in the thrombin production. The inhibition of factor Xa by Rivaroxaban is concentration dependent and rivaroxaban also inhibits its amidolytic activity [16–18]. The affinity of Rivaroxaban is > 10,000 times more towards human factor Xa than factor Xa of any other species. Further it has been demonstrated that during post rivaroxaban treatment in in vitro studies there is prolongation of initial phase of thrombin production and reduction thrombin production during propagation phase [19].

The interaction between BSA and rivaroxaban has not been studied till date even though several pharmacokinetic and pharmacodynamics studies have been performed on this drug. The study of these interactions (biophysical) help in understanding the behavior of drug molecules in vivo [20–25]. A huge amount of data can be obtained regarding the structural details of drugs and therapeutic capabilities with the help of these interaction studies. The level of binding of drug ligand to the protein is important for studying its distribution and/or elimination from body.

In this research paper multi-spectroscopic approaches were used to study biophysical interaction of albumin and rivaroxaban. These approaches included spectrofluorometric quenching experiments along with molecular docking studies. This study will provide further understanding regarding the PK/PD behavior of the rivaroxaban.

## Results and discussion

### UV absorption spectra of BSA

**Fig. 1:**
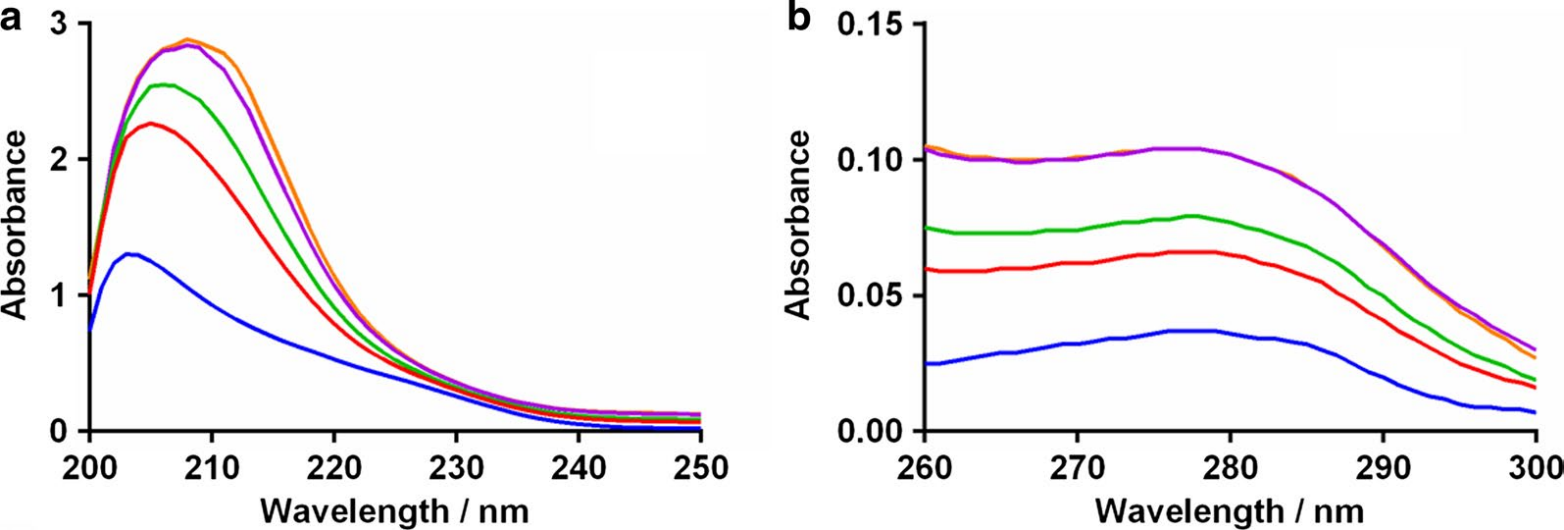
UV spectra of BSA in the presence of rivaroxaban. **a** Represents the spectra at 210 nm and **b** at 280 nm

To explore the changes in the structure and conformation of rivaroxaban and BSA complex UV absorption spectroscopy was utilized [26]. The UV spectra for BSA alone and its complex with rivaroxaban are presented in Fig. 1. In Fig. 1a, b two absorption bands exist for BSA in presence of rivaroxaban. The strong band occurs at near 210 (Fig. 1a) and weak band at near 280 nm (Fig. 1b). The conformational framework of BSA is characterized by the absorption band near 210 nm whereas, π → π transition due aromatic amino acids represent the band at 280 nm. With increasing concentration of rivaroxaban the absorption intensities also increased. The development of complex between BSA and rivaroxaban is indicated because of red shift at 210 nm and blue shift at 280 nm.

### Fluorescence quenching of BSA

**Fig. 2:**
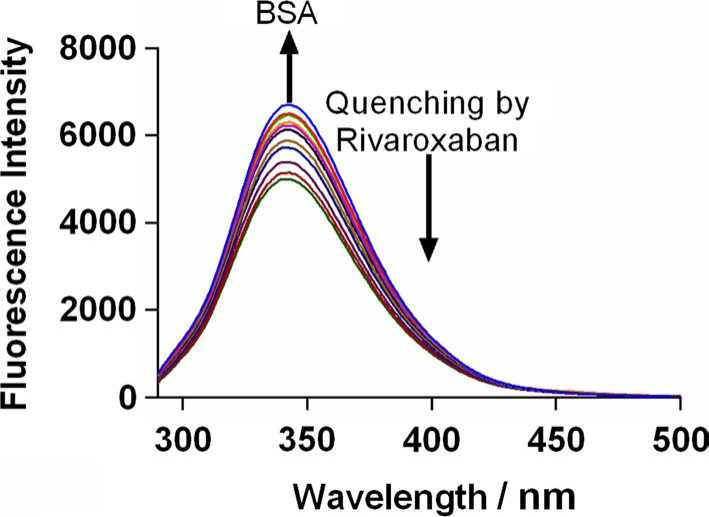
The fluorescence quenching spectra of BSA in the presence of rivaroxaban at 25 °C, λex = 280 nm, and λem = 340 nm

Fluorescence quenching studies to explore the binding interaction of drug ligands with proteins is considered as the best methodology [27]. Figure 2 represents the fluorescence spectra of BSA alone as well as in combination with different concentrations of rivaroxaban. The FI showed a decrease with increasing concentrations of rivaroxaban with slight alteration in the λemission. This indicated that there was some alteration in the micro-environment of the fluorophore Trp-213 upon interaction of BSA and rivaroxaban [28].

### Analysis of fluorescence quenching and mechanism

The quenching processes can be dynamic quenching and static quenching. In static quenching, the complex formed between the ligand and the albumin is non-fluorescent. While as in dynamic quenching there occurs a molecular collision amongst the drug ligands and albumin during the lifetime excited state.

At higher temperatures the dynamic quenching constant is increased because of higher diffusion coefficient values. This increased diffusion coefficient augments the electron transfer processes in case of dynamic quenching. In static quenching the quenching constant behaves in opposite to that of dynamic quenching at elevated temperatures because of the instability of ground state complex. The mechanism of fluorescence quenching can be evaluated by Stern–Volmer equation:

$$\frac{F}{{F_{0} }} = 1 + K_{sv} \left[ Q \right] = 1 + K_{q} \tau_{0} \left[ Q \right]$$

The FI of BSA in presence and absence of the quencher are designated by F and F_0_; K_sv_ is Stern–Volmer constant; [Q] is quencher concentration; K_q_ is bimolecular quenching rate constant; τ_0_ is fluorophore’s lifetime without quencher, and is assigned to be 10^−8^ for a biopolymer.

**Fig. 3:**
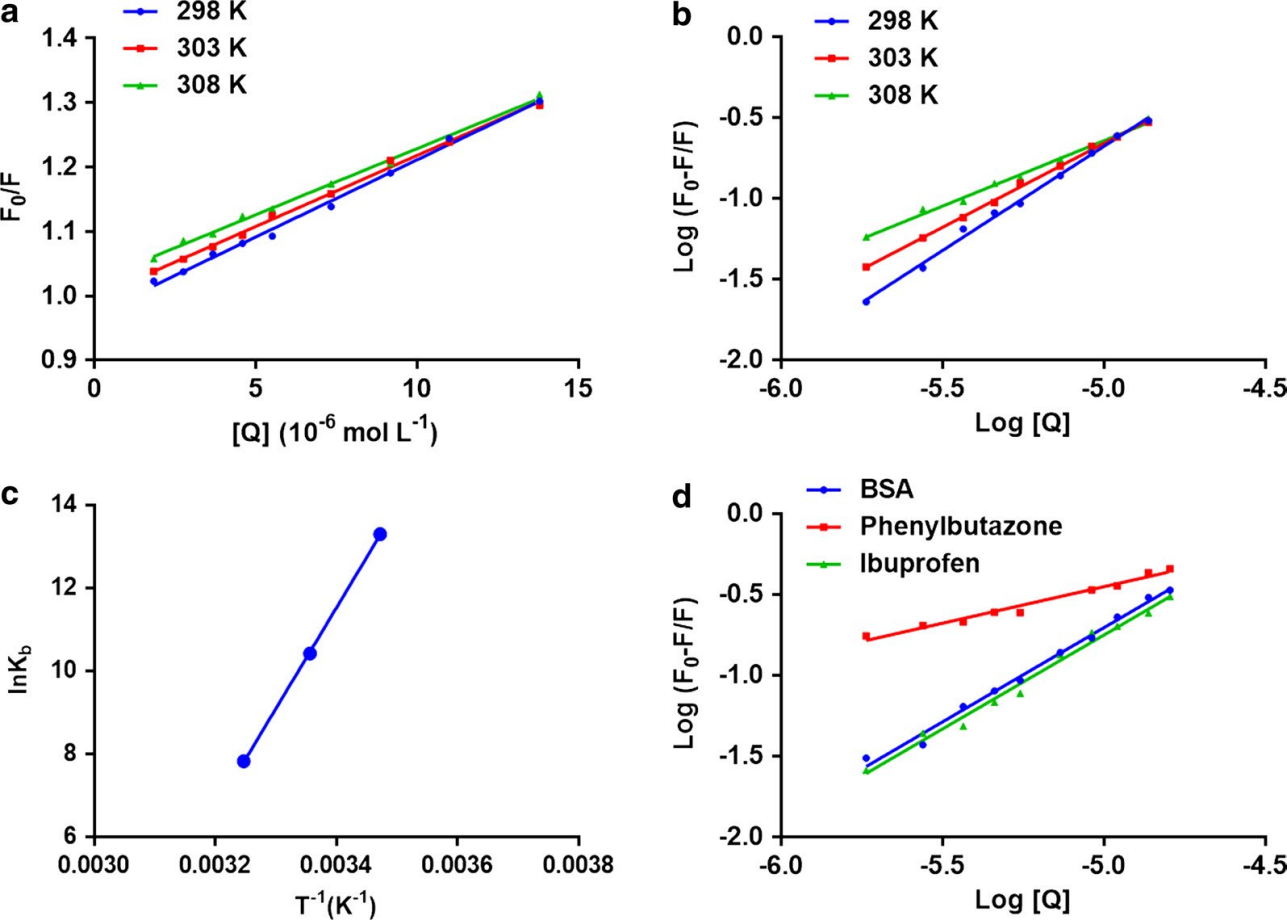
**a** The Stern–Volmer curves for the quenching of BSA by rivaroxaban at 298/303/308 K. **b** The plot of log[(F_0_ − F)/F] versus log[Q] for quenching process of rivaroxaban with BSA at 298/303/3008 K. **c** Van’t Hoff plots for the binding interaction of rivaroxaban with BSA. **d** The plot of log[(F_0_ − F)/F] versus log[Q] for quenching process of rivaroxaban with BSA in presence of site markers phenylbutazone and ibuprofen at 298 K

The value for K_q_ also helps in determination of mechanism of quenching involved. The maximum scattering collision quenching rate constant attained by quencher-BSA complex is 2 × 10^10^ M^−1^ S^−1^. Table 1 along with Fig. 3a shows that the K_sv_ value increases with increased temperatures indicating a dynamic quenching process. Also, the values obtained for K_q_ are more than the values of 2 × 10^10^ M^−1^ S^−1^ indicating formation of non-fluorescent complex between rivaroxaban and BSA. The dissimilarity among the different types of quenching behaviors could be explained with changes in the UV–visible spectrum of BSA. The absorption spectra for the quencher is unaffected in case of dynamic quenching as it influences only the excitation state of the quencher. In static quenching the complex is formed among the BSA and ligand, resulting in the change of the absorbance spectra of BSA molecule. As discussed earlier a complex is formed amongst the BSA molecule and rivaroxaban (Fig. 1) inferring that fluorescence quenching is primarily due to this complex formation (static quenching) [29].

**Table 1:** Stern–Volmer quenching constants (K_SV_) and bimolecular quenching rate constant (Kq) for the binding of rivaroxaban to BSA at three variable temperatures

T (K)	R	Ksv ± SD × 10^4^ (L mol^−1^)	Kq × 10^12^ (L mol^−1^ s^−1^)
298	0.9933	2.25 ± 0.21	2.25
303	0.9921	2.33 ± 0.19	2.33
308	0.9973	2.43 ± 0.15	2.43

### Binding constant and binding modes

In static quenching it is assumed that several binding sites (n) are available on the BSA for binding the drug. The binding constant (K_b_) and n are calculated by using double log regression curve Fig. 3b [30]. The intercept and slope of the plotted curve is used to calculate K_b_ and n Table 2

$$\log \frac{{\left( {F_{0} - F} \right)}}{F} = \log K_{b} + n \log \left[ Q \right]$$

**Table 2:** Binding and thermodynamic parameters of binding between rivaroxaban and BSA

T (K)	R	Log K_b_ ± SD	K_b_ (L mol^−1^)	n	∆G (kJ mol^−1^)	∆H (kJ mol^−1^)	∆S (J mol^−1^ K^−1^)
298	0.9914	5.12 ± 0.09	1.32 × 10^5^	1.1	− 24.67	− 126	− 340
303	0.9818	4.25 ± 0.14	1.82 × 10^4^	0.98	− 22.97		
308	0.9895	3.64 ± 0.11	4.37 × 10^3^	0.85	− 21.27		

The high K_b_ suggests a very strong binding interaction between rivaroxaban and BSA inferring low free plasma concentration of rivaroxaban in vivo. The value of n of BSA at all three studied temperatures is approximately equivalent to 1 as fractional binding sites don’t occur and no < 1 binding site can be present suggesting only one binding site for rivaroxaban. Also, a lowering in binding site number was observed at higher temperature and can be attributed to the fact that at higher temperatures the molecules are disordered and undergo fast vibrations and can have higher diffusion coefficients which may lead to instability of rivaroxaban–BSA complex.

Further, the value of the correlation coefficient (r^2^) at temperatures of 298, 303 and 308 K were (> 0.99) suggesting that rivaroxaban and BSA interaction precisely followed double logarithm regression based site-binding model. Site specific probes (phenylbutazone and ibuprofen) were used to establish the binding sites of rivaroxaban on BSA. The concentration of BSA and site specific probe were kept constant, and equimolar concentration for both of them were used whereas the concentration of rivaroxaban was varied. The fluorescence spectra were obtained at 25 °C (room temperature) at (λexcitation = 280 nm). The binding constant (Kb) attained under these conditions were 0.63 × 10^2^ for the rivaroxaban and BSA (with phenylbutazone as probe) and 1.13 × 10^5^ (with ibuprofen). The binding constant for rivaroxaban and BSA complex was 1.32 × 10^5^. The results showed a reduction in the binding constants with the presence of probes. The lowest binding constant was obtained with phenylbutazone as site probe suggesting site I (sub-domain IIA) as the principal binding site for rivaroxaban (Fig. 3d). However, some binding also occurred at site II (sub-domain IIIA) with a decrease in the binding constant when ibuprofen was used as a probe specific for site II [31].

### Thermodynamic parameters and binding forces

The protein binding of drugs is due to some kind of binding forces which include hydrogen bonding interaction, van der Waals forces, electrostatic interaction and hydrophobic interaction. The type of forces involved in these binding interactions are determined by the signs and amounts of thermodynamic parameters that are calculated by following equation (van’t Hoff equation):

$$\ln Kb = - \frac{{\Delta H^{0} }}{RT} + \frac{{\Delta S^{0} }}{R}$$

$$\Delta G^{0} = \Delta H^{0} - T\Delta S^{0} = - RT\ln Kb$$

where, ∆G^0^ is change of Gibbs free energy; ∆H^0^ is change of enthalpy and ∆S^0^ is change of entropy; R is gas constant and K_b_ the binding constant at different temperatures used in this study. The involvement of van der Waals forces and/or hydrogen bonding is suggested by negative (−) values in ∆H^0^ and ∆S^0^ whereas positive values in ∆H^0^ and ∆S^0^ suggest a hydrophobic interaction. ∆H^0^ value approximating zero and (+) ∆S^0^ suggests electrostatic interaction forces [31, 32]. The BSA rivaroxaban van’t Hoff plot is represented in Fig. 3c and the enthalpy and entropy as well as gibbs free energy values are presented in Table 2. The negative value of ∆G^0^ suggests that the rivaroxaban and BSA binding was spontaneous. The negative values for ∆H^0^ and ∆S^0^ showed that the interaction of BSA with Rivaroxaban is mainly enthalpy driven. The negative value of entropy suggests unfavorable binding process like van der Waals interactions and hydrogen bonding in interaction of rivaroxaban to BSA.

### Synchronous fluorescence spectroscopy of BSA and rivaroxaban complex

**Fig. 4:**
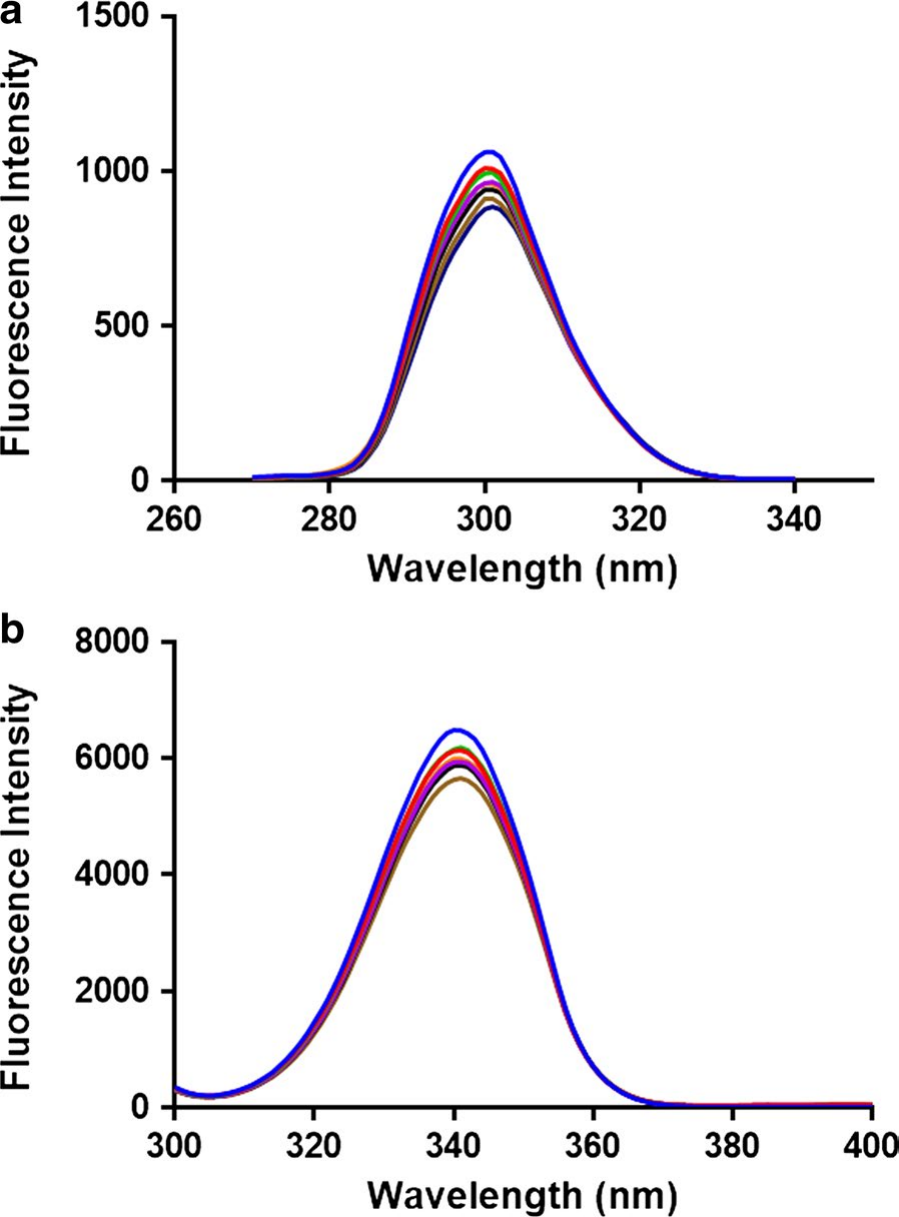
Synchronous fluorescence spectroscopy of BSA at 298 K **a** ∆λ = 15 nm and **b** ∆λ = 60 nm

**Fig. 5:**
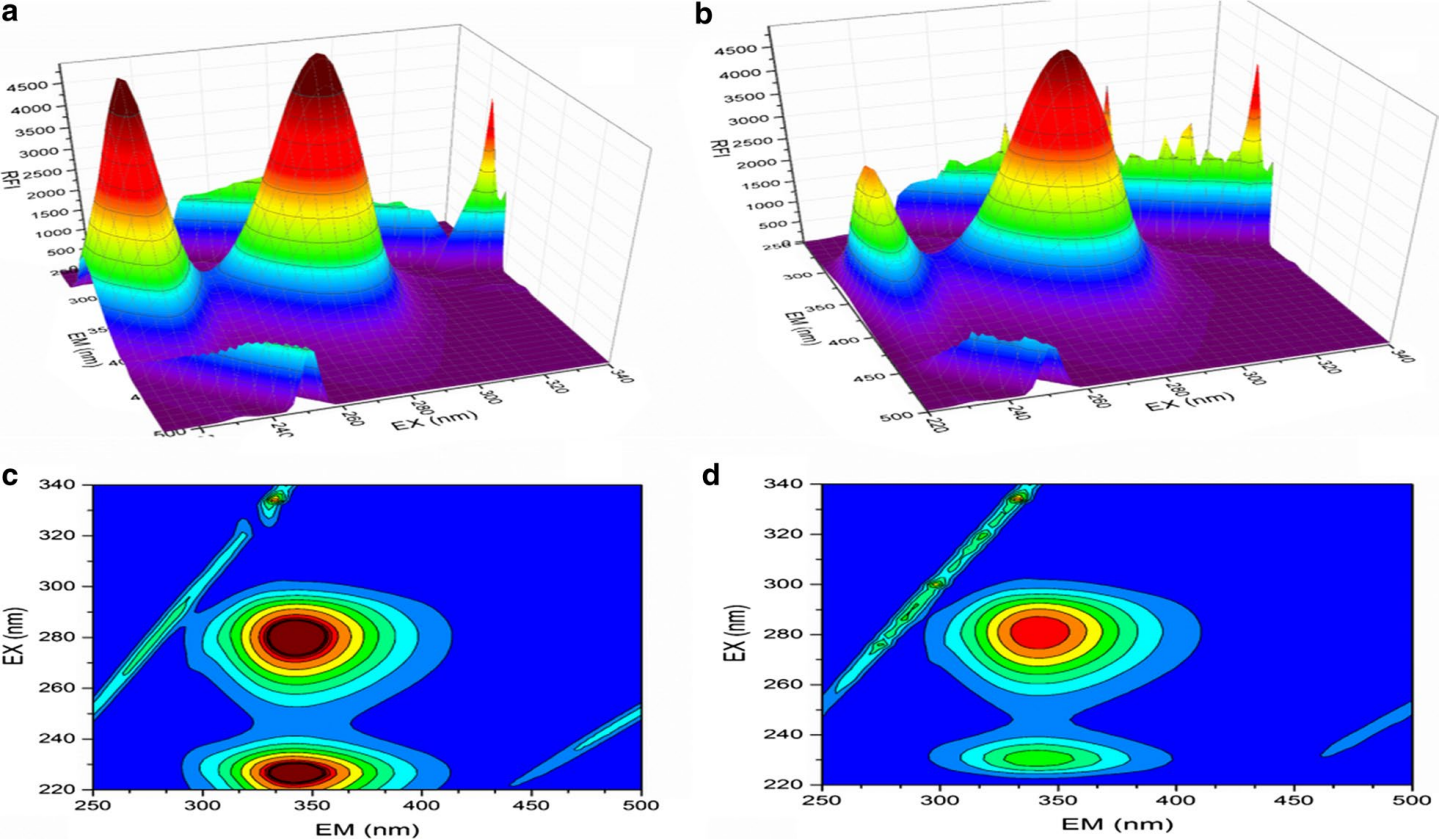
Three-dimensional fluorescence (3D) spectra and contour spectra of BSA (**a**, **c**) and BSA–rivaroxaban (**b**, **d**) complex BSA

The secondary structure formed post BSA–rivaroxaban interaction was studied with help of SF spectroscopy [33]. SF spectroscopy provides us with the evidence about microenvironment surrounding the chromophores. The scanning intervals of ∆λ = 15 nm provide specific information about the tyrosine residue and ∆λ = 60 nm provide information about tryptophan residues. In case a shift occurs in the maximum λemission of the BSA, it indicates an alteration in the micro-environmental polarity of tyrosine or tryptophan or both of them. Different spectra were obtained for BSA alone and with rivaroxaban and the results showed a decreased FI upon addition of rivaroxaban Fig. 4. There was a shift of 1 nm at both ∆λ = 15 nm and ∆λ = 60 nm suggests a modification in the micro-environmental vicinity of tyrosine and tryptophan upon binding to rivaroxaban. 3D (3-dimensional) spectra for BSA were also obtained in presence/absence of rivaroxaban [34]. Two peaks were observed in the BSA namely 1 and 2. Peak 2 (λex/λem: 275.0/340.0 nm) is because of existence of tryptophan and tyrosine residues. Figure 5a represents the FI in absence of rivaroxaban and Fig. 5b indicates a decrease in the FI of BSA post addition of rivaroxaban because of quenching of its fluorescence by rivaroxaban. The result (Table 2) indicates lesser polar microenvironment of both tryptophan and tyrosine residues and the hydrophobic amino acids might be buried deep within hydrophobic pockets. Further the less polar environment suggests that rivaroxaban binds to the hydrophobic pocket in BSA and upon addition changes the conformational polarity of the hydrophobic microenvironment of BSA.

The fluorescence spectral features of the polypeptides present in BSA are represented by peak 1 (λex/λem: 225.0/340.0 nm) and are due to π–π* transition of the polypeptide structures (C=O) [35, 36]. There was a steep decline in the intensity of peak after addition of rivaroxaban and the FI decreased as indicated in the Table 3. As evident in the contour plot (Fig. 5) the lower portion of the spectra was sparse post addition of rivaroxaban compared to BSA alone indicating that there was conformation change BSA post rivaroxaban addition.

**Table 3:** Three dimensional fluorescence spectra parameters for BSA and BSA–rivaroxaban complex

System	Parameters	Peak 1	Peak 2
BSA	Peak position (λex/λem, nm)	226.0/342.0	282.0/342.0
	Fluorescence intensity	5527	5573
	Stokes shift Äë (nm)	116	60
BSA–rivaroxaban	Peak position (λex/λem, nm)	230.0/342.0	282.0/3420
	Fluorescence intensity	2946	4924
	Stokes shift ∆λ (nm)	112	60

### Molecular simulation studies

**Fig. 6:**
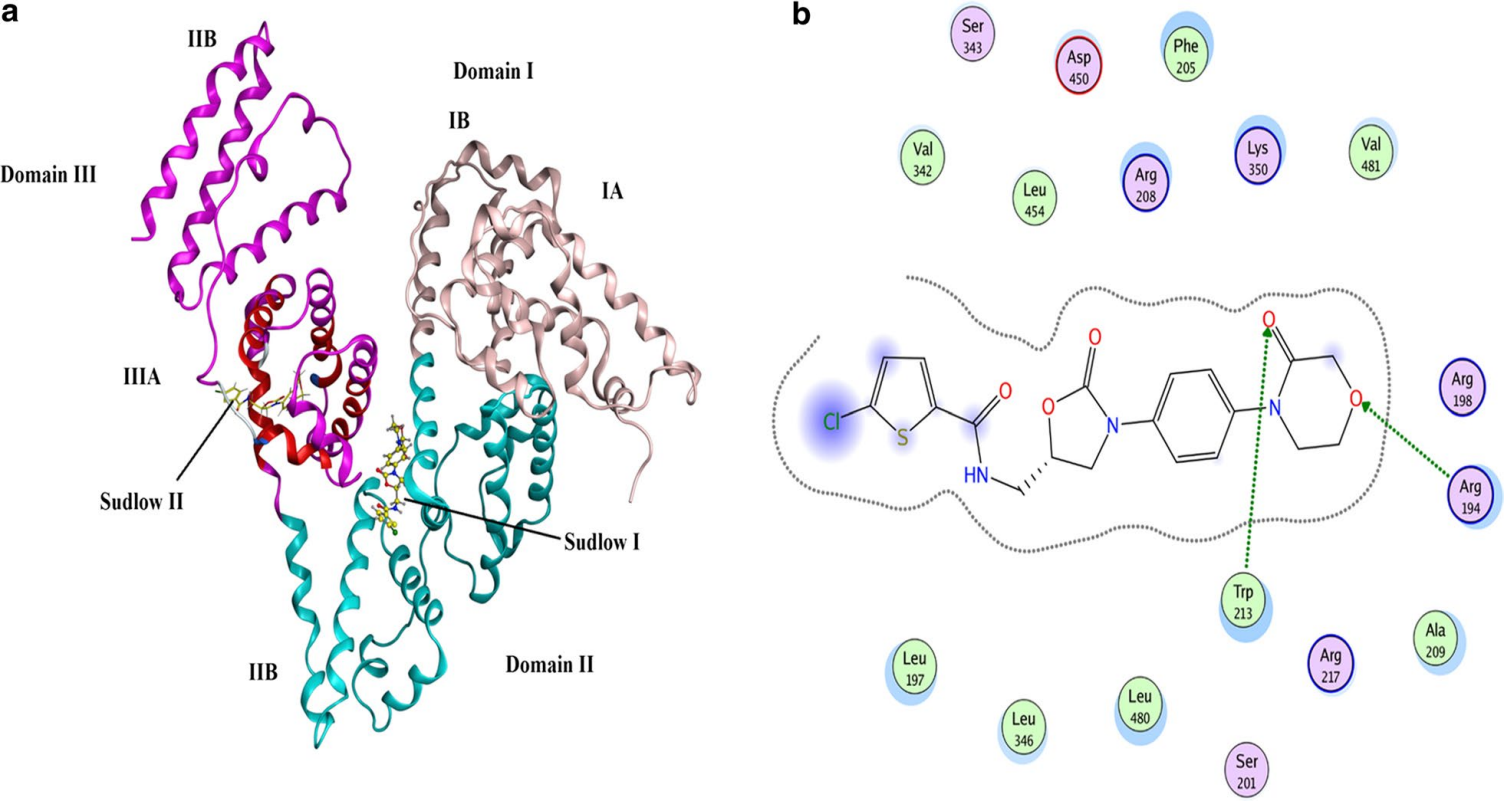
**a** The docking conformation of rivaroxaban–BSA complex with lowest energy. **b** The amino acid residues surrounding rivaroxaban

To further understand the BSA rivaroxaban interaction the molecular docking studies were performed. The molecular docking studies complimented with the UV spectroscopic and fluorescence results. In the docking analysis the rivaroxaban was docked with BSA to establish the favored binding site and the binding mode. BSA protein has two ligand binding sites (Site I/Site II) and represent the hydrophobic binding grooves of sub-domains IIA IIIA respectively. The best conformation of rivaroxaban and BSA is presented in Fig. 6a. As presented in Fig. 6 the rivaroxaban binds to both site I/II of sub-domain IIA/IIIA pocket in domain II and III of BSA. These docking and spectroscopic results are in agreement with each other since the microenvironment of both amino acid residues (tyrosine and tryptophan) were altered upon addition of rivaroxaban to BSA. Figure 6b demonstrates the hydrogen bonding between rivaroxaban and BSA. At site I rivaroxaban formed hydrogen bonds with ARG-194 and TRP-213 residues and was encircled by ARG- 208, VAL-342, LEU-454, PHE-205, ARG-198, ARG-194, ARG- 217, LYS-350, ALA-209, LEU-197, LEU-346, LEU-480 and VAL-481. On site II rivaroxaban formed hydrogen bonds with LYS-413, TYR-410 and CYS-437 and was encircled by GLN 393, LEU-452, LEU-386, LEU-406, LEU-429, GLY-433, SER-488, THR448, VAL-432, GLN-389 and ARG-409 with the binding energies for the BSA–rivaroxaban complex as − 32.38 kJ mol^−1^ at site I and 25.89 kJ mol^−1^ at site II. The experimental binding constant value at 300 K was found to be − 24.67 kJ mol^−1^ and is similar to the binding constant value obtained theoretically.

## Conclusion

Rivaroxaban binds mainly to site I (sub-domain IIA) of the BSA and a complex is formed between the two molecules with the inherent fluorescence of BSA quenched by rivaroxaban. Further, rivaroxaban also binds to the Site II (sub-domain IIIA) as indicated during the molecular docking analysis. A single binding site was observed in the BSA–rivaroxaban complex and the binding constants indicated that their binding is quite strong to be highly bound in plasma. These results corroborated with site specific probes which indicated site I (sub domain IIA) as the principal binding site for rivaroxaban. The thermodynamic studies showed that interaction between BSA and rivaroxaban is mainly enthalpy driven with involvement of van der Waals interactions and the hydrogen bonding.

## Experimantal

### Chemical and reagents

The BSA was purchased from Sisco Research Laboratories India, rivaroxaban, phenylbutazone and ibuprofen was procured by from National Scientific Company; Saudi Arabia. The chemicals used for the study were of analytical grade.

Solutions of BSA, rivaroxaban, phenylbutazone and ibuprofen were prepared according to their molecular weights. The working standards of BSA (1.5 µM) was prepared in phosphate buffer (pH 7.40). The stock of rivaroxaban (2.3 × 10^−3^ M) was prepared with the addition of suitable amount of standard rivaroxaban in 500 µL dimethyl sulphoxide with final volume made up by phosphate buffer. The working standards were in the range between 1.6 × 10^−6^ and 8 × 10^−6^ prepared from the stock. Similarly, the stocks of phenylbutazone and ibuprofen were prepared by dissolving them in methanol with further dilutions in phosphate buffer. Water-IV (Elga Purelab FLEX type-IV; Elga Lab Water UK) was used in preparation of the stocks and all working standards.

### UV spectra measurements

The UV spectrophotometer, UV-1800 from Shimadzu, Japan was used for all the spectrophotometric measurements. The measurements were done for the BSA alone as well as in presence of varying rivaroxaban concentrations. All the spectra were obtained at room temperature.

### Fluorescence measurements

The fluorescence spectra were obtained from JASCO FP-8200 (Easton, USA) spectrofluorometer at three different temperatures (298, 303 and 308 K) at wavelength of 280 and 340 nm for excitation and emission respectively. The standard solutions of similar concentration of BSA fixed (1.5 × 10^−6^ M) and varying concentration of rivaroxaban (1.6 × 10^−6^ to 8 × 10^−6^ M) were mixed in the 1:1 *v/v* ratio in different 10 mL volumetric flasks. The final concentration for the analysis were BSA 0.75 × 10^−6^ M and rivaroxaban ranged from 0.8 × 10^−6^ to 4 × 10^−6^ M. The measurements were repeated three times and the final mean of the three readings were taken. The existence of inner filter effect results in decreased fluorescence intensity. In case, a compound present in the fluorescence detection system shows absorption in the UV region at its excitation or emission wavelength can result in inner filter effect. The fluorescence intensities were corrected for studying the interaction between rivaroxaban and BSA using the following equation [20]:

$$Fcor = Fobs \times e^{{\left( {Aex + Aem} \right)/2}}$$

F_*cor*_ (corrected fluorescence), and F_*obs*_ (observed fluorescence), A^ex^ (rivaroxaban absorption at excitation wavelength) and A^*em*^ (rivaroxaban absorption at emission wavelength).

### Synchronous fluorescence (SF) measurement

The rivaroxaban and BSA solutions synchronous fluorescence spectra were attained using the JASCO spectrofluorometer at 25 °C (room temperature) with altered scanning intervals of ∆λ (∆λ = λ_em_ − λ_ex_). The properties of tyrosine and tryptophan residues residue were characterized at ∆λ = 15 nm and at ∆λ = 60 nm respectively.

#### Molecular docking

The molecular docking analysis were performed to evaluate the interaction behavior of rivaroxaban with BSA. The docking was performed on Molecular Operating Environment (MOE-2014). Chemical structure of rivaroxaban was drawn in the MOE software whereas the crystal structure of BSA (PDB ID 4OR0) was imported from Protein Data Bank (http://www.rcsb.org). The resulting structures were minimized using MMFF94x force-field reaction with following electrostatics Din = 1, Dout = 80. To all the atoms a tether (flat bottom) of 10.0 kcal mol^−1^ and 0.25 Å was applied. RMSD parameters (root mean square deviation) was utilized for the selection of the most appropriate interaction of BSA with rivaroxaban.

## Abbreviations

FI: fluorescence intensity
PK/PD: pharmacokinetics and pharmacodynamics
BSA: bovine serum albumin
HSA: human serum albumin
